# Distinguishing patients with laboratory-confirmed chikungunya from dengue and other acute febrile illnesses, Puerto Rico, 2012–2015

**DOI:** 10.1371/journal.pntd.0007562

**Published:** 2019-07-22

**Authors:** Luisa I. Alvarado, Olga D. Lorenzi, Brenda C. Torres-Velásquez, Tyler M. Sharp, Luzeida Vargas, Jorge L. Muñoz-Jordán, Elizabeth A. Hunsperger, Janice Pérez-Padilla, Aidsa Rivera, Gladys E. González-Zeno, Renee L. Galloway, Mindy Glass Elrod, Demetrius L. Mathis, M. Steven Oberste, W. Allan Nix, Elizabeth Henderson, Jennifer McQuiston, Joseph Singleton, Cecilia Kato, Carlos García-Gubern, William Santiago-Rivera, Robert Muns-Sosa, Juan D. Ortiz-Rivera, Gerson Jiménez, Vanessa Rivera-Amill, Doris A. Andújar-Pérez, Kalanthe Horiuchi, Kay M. Tomashek

**Affiliations:** 1 Ponce Health Sciences University /Ponce Research Institute, Saint Luke's Episcopal Hospital, Ponce, Puerto Rico, United States of America; 2 Dengue Branch, Division of Vector-Borne Diseases, Centers for Disease Control and Prevention (CDC), San Juan, Puerto Rico, United States of America; 3 Division of Global Health Protection, Centers for Disease Control and Prevention (CDC), Kenya, Africa; 4 Bacterial Special Pathogens Branch, Zoonoses and Select Agent Laboratory, CDC, Atlanta, Georgia, United States of America; 5 Polio and Picornavirus Laboratory Branch, Division of Viral Diseases, National Center for Immunization and Respiratory Diseases, CDC, Atlanta, Georgia, United States of America; 6 Rickettsial Zoonoses Branch, Division of Vector-Borne Diseases, CDC, Atlanta, Georgia, United States of America; 7 Saint Luke’s Episcopal Hospital, Guayama, Puerto Rico, United States of America; 8 Office of the Director, Division of Vector-Borne Diseases, CDC, Fort Collins, Colorado, United States of America; 9 National Institute of Allergy and Infectious Diseases, National Institutes of Health, Bethesda, Maryland, United States of America; University of Pittsburgh, UNITED STATES

## Abstract

Chikungunya, a mosquito-borne viral, acute febrile illness (AFI) is associated with polyarthralgia and polyarthritis. Differentiation from other AFI is difficult due to the non-specific presentation and limited availability of diagnostics. This 3-year study identified independent clinical predictors by day post-illness onset (DPO) at presentation and age-group that distinguish chikungunya cases from two groups: other AFI and dengue. Specimens collected from participants with fever ≤7 days were tested for chikungunya, dengue viruses 1–4, and 20 other pathogens. Of 8,996 participants, 18.2% had chikungunya, and 10.8% had dengue. Chikungunya cases were more likely than other groups to be older, report a chronic condition, and present <3 DPO. Regardless of timing of presentation, significant positive predictors for chikungunya versus other AFI were: joint pain, muscle, bone or back pain, skin rash, and red conjunctiva; with dengue as the comparator, red swollen joints (arthritis), joint pain, skin rash, any bleeding, and irritability were predictors. Chikungunya cases were less likely than AFI and dengue to present with thrombocytopenia, signs of poor circulation, diarrhea, headache, and cough. Among participants presenting <3 DPO, predictors for chikungunya versus other AFI included: joint pain, skin rash, and muscle, bone or back pain, and absence of thrombocytopenia, poor circulation and respiratory or gastrointestinal symptoms; when the comparator was dengue, joint pain and arthritis, and absence of thrombocytopenia, leukopenia, and nausea were early predictors. Among all groups presenting 3–5 DPO, pruritic skin became a predictor for chikungunya, joint, muscle, bone or back pain were no longer predictive, while arthritis became predictive in all age-groups. Absence of thrombocytopenia was a significant predictor regardless of DPO or comparison group. This study identified robust clinical indicators such as joint pain, skin rash and absence of thrombocytopenia that can allow early identification of and accurate differentiation between patients with chikungunya and other common causes of AFI.

## Author summary

Chikungunya is an acute febrile illness (AFI), caused by the chikungunya virus (CHIKV), that is transmitted by mosquitoes. Patients with chikungunya exhibit joint, muscle, or bone pain, and may also have skin rash, red eyes (conjunctiva), and red swollen joints (arthritis). Up to 20% of cases develop long lasting arthritis, fatigue or psychiatric conditions. We used data from our prospective study to identify signs and symptoms that predict chikungunya. We enrolled 8,996 AFI patients and tested for CHIKV, dengue viruses 1–4 (DENV 1–4), and other pathogens. A pathogen was detected in 55% of participants; 18.2% had CHIKV and 10.8% had DENV 1–4 infections. This study compared the clinical presentation of chikungunya with that of all other AFI and dengue alone. Regardless of timing of presentation, significant predictors of chikungunya were joint pain, muscle, bone or back pain, and red conjunctiva when compared to other AFI, and arthritis, joint pain, skin rash, any bleeding, and irritability, when dengue was the comparator group. Chikungunya patients were less likely than AFI and dengue patients to have low platelets, signs of poor circulation, diarrhea, headache, and cough. By enrolling febrile patients at presentation, we identified unbiased predictors of chikungunya. These findings can assist physicians to clinically diagnose chikungunya and initiate proper patient management.

## Introduction

Chikungunya is an acute febrile illness (AFI) caused by an *alphavirus*, chikungunya virus (CHIKV) [1]. CHIKV spreads from viremic humans to *Aedes* species mosquitoes that can transmit the virus to other humans when taking a blood meal. CHIKV can also be transmitted from an infected mother to her child during pregnancy or parturition [2]. Transmission via infected donor blood products and organs is a theoretical risk; however, no cases of transfusion-transmitted or organ transplant-transmitted CHIKV infection have been reported [3].

After an incubation period of typically 3–7 days following the bite of a CHIKV-infected mosquito, most people become symptomatic [4]. Symptoms include high fever, bilateral symmetric joint pain, myalgia, arthritis, maculopapular rash, conjunctivitis, headache, and nausea or vomiting. Clinical laboratory findings can include lymphopenia, thrombocytopenia, elevated creatinine, and elevated hepatic transaminases. Rare complications include uveitis, retinitis, myocarditis, hepatitis, nephritis, bullous skin lesions, meningoencephalitis, myelitis, Guillain-Barré syndrome, and cranial nerve palsies [5–7]. Mortality is thought to be a rare outcome [8]. Persons at risk for severe disease include neonates exposed intrapartum, adults >65 years old, and people with chronic medical conditions [9, 10]. While the acute illness typically resolves by the end of the third week, some people have a post-acute phase with arthritis, neuropathy, and neuropsychiatric conditions that may last through the third month [10–17]. Chronic musculoskeletal symptoms, which are likely mediated by inflammation [13] potentially resulting from viral persistence [18–21], may recur or persist more than 3 months after the acute phase of the illness.

While most people are thought to be immune after a single infection, currently, there is no vaccine to prevent CHIKV infection and no specific antiviral treatment for patients with chikungunya, although several vaccines and therapeutic candidates are under development [22]. Symptomatic treatment with analgesics and non-steroidal anti-inflammatory drugs (NSAIDs) is recommended for those with fever and joint symptoms [1]. Aspirin is not recommended due to the increased risk of bleeding, and corticosteroids are not recommended in the acute and post-acute phase as they may cause immunosuppression that may worsen the clinical course [23]. NSAIDs, corticosteroids, and methotrexate are the recommended treatments for chronic chikungunya arthritis [17, 23–25].

The clinical diagnosis of chikungunya may be complicated if the patient resides in or has recently traveled to a dengue endemic area. Early in the clinical course, chikungunya cases may be difficult to distinguish from cases of dengue, adenoviral disease, influenza, leptospirosis, Zika, and malaria. Testing blood for viral RNA by reverse transcription-polymerase chain reaction (RT-PCR) [26] or anti-CHIKV antibodies by IgM antibody capture enzyme-linked immunosorbent assay (MAC-ELISA) [27] allows for a definitive diagnosis. During outbreaks and in resource poor settings, diagnosis often relies on the identification of clinical features consistent with the World Health Organization (WHO) case definition [26]. However, the sensitivity and specificity of this definition is not known and may vary by timing of presentation and age of the patient [27, 28].

Laboratory diagnosis, while not often feasible, is essential for patient care and improving public health. Identifying AFI patients who have chikungunya is important for patients who develop post-acute and/or chronic disease. It can also improve patient outcomes by enabling more timely assessment of patients with other AFIs that require early administration of an antibiotic or antiviral drug, or specific anticipatory guidance. Also, identification of patients with CHIKV infection early, while the patient is still febrile (and viremic), may help limit further transmission of CHIKV within households and communities. In this manuscript, we describe clinical predictors of RT-PCR positive chikungunya cases by the timing of presentation and age compared to two groups: all other AFI cases (CHIKV-negative), and RT-PCR positive dengue cases. To do so, we utilized the first three years of data collected from an ongoing clinical study in which patients presenting to the hospital emergency department with AFI were enrolled and tested for evidence of infection with CHIKV, dengue virus 1–4 (DENV-1–4), and 20 other pathogens.

## Materials and methods

### Ethics statement

Before enrollment, informed consent was administered by study staff in accordance with Puerto Rico law (Article 13, Section 13, Regulation 7617 of the Office of Patient Ombudsman, Act #194). Specifically, written informed consent was obtained from eligible adults ≥21 years old and emancipated minors 14–20 years old. Written informed consent was obtained from parents of minors ≤ 20 years old. Written informed assent was obtained from non-emancipated minors 14–20 years old and verbal informed assent was obtained from children 7–13 years old. The Institutional Review Boards at the Centers for Disease Control and Prevention (CDC) and Ponce Medical School Foundation (PMSF) approved the study protocol.

### Study population

The study was conducted in southern Puerto Rico at Saint Luke’s Episcopal Hospital (SLEH), a tertiary care teaching hospital in Ponce with more than 54,000 annual Emergency Department (ED) visits, and SLEH–Guayama, a secondary acute care hospital in Guayama with 40,000 annual ED visits. Together, the hospitals provide clinical services to about 600,000 residents of neighboring municipalities [29].

### Study enrollment and procedures

Study procedures were previously described [29]. In brief, enrollment was conducted between May 7, 2012 and May 6, 2015 at SLEH–Ponce and February 1, 2013 and May 6, 2015 at SLEH–Guayama. Consenting patients presenting to the ED or as a direct hospital admission were enrolled if they had a fever defined by a body temperature of ≥38.0°C (oral) or ≥38.5°C (axillary), or history of fever for seven or fewer days. After informed consent was administered, demographic information, clinical features, exposure history, and history of chronic disease were collected using a structured questionnaire. A physician examined the participant and recorded the clinical diagnosis. The following pre-existing conditions were collected at enrollment in the case review form: diabetes, high blood pressure, coronary heart disease, high cholesterol, asthma, chronic obstructive pulmonary disease, cancer, immunodeficiency, chronic kidney disease, chronic liver disease, thyroid disease, and sickle cell disease. Study participants returned 7–30 days post-illness onset (DPO) to provide convalescent specimens and complete a questionnaire recording healthcare services received and signs and symptoms experienced since enrollment.

### Specimen collection

At enrollment, blood, urine and oro-nasopharyngeal specimens were collected. Convalescent blood and urine were collected >7 DPO. Sample collection procedures have been previously described [29].

### Laboratory diagnostics

Molecular diagnostic testing for CHIKV, DENV 1–4, influenza A and B viruses, and 12 other respiratory viruses including adenovirus, human respiratory syncytial virus, human metapneumovirus, parainfluenza virus 1–4, human enterovirus/rhinovirus, and four human coronaviruses (229E, OC43, NL63 and HKU1), was performed as described previously [29, 30]. Serum specimens collected ≤6 DPO were tested by a DENV-serotype specific real-time RT-PCR [31, 32], and those collected ≥4 DPO were tested by an anti-DENV MAC-ELISA (InBios International, Inc., Seattle, WA)[33–35]. Beginning in May 2014, specimens collected ≤6 DPO were tested by CHIKV-specific, real-time RT-PCR [36], and those collected ≥6 DPO were tested by anti-CHIKV MAC-ELISA [33]. Serum specimens collected ≤3 DPO were tested by a pan-enterovirus real-time RT-PCR assay that targets the viral protein 1 (VP1) region [37]. Paired serum specimens from enrollment and follow-up visit were tested by microscopic agglutination test (MAT) for *Leptospira* spp. [38] and by indirect hemagglutination assay (IHA) for *Burkholderia pseudomallei* [39], according to an algorithm that was previously described [29]. The first 250 patients with *Leptospira spp*. and *B*. *pseudomallei* negative specimens and for which paired specimens were available were tested by Indirect Fluorescent Assay (IFA) for *Rickettsia spp*., *Ehrlichia spp*., and *Coxiella spp*. Whole blood and/or acute serum from cases with a reactive IFA were assessed for *C*. *burnetii*, *R*. *rickettsii*, *R*. *typhi*, *and/or E*. *chaffeensis* DNA by PCR [29].

### Clinical definitions

Leukopenia was defined as a white blood cell count ≤5,000 cells/μL. Thrombocytopenia was defined as a platelet count ≤100,000/μL. Severe hemoconcentration was defined by a hematocrit ≥20% above the U.S. population mean hematocrit for age and sex, and moderate hemoconcentration was defined by a hematocrit >97.5^th^ percentile for age and sex to less than the cut-off for severe hemoconcentration [40]. A skin bleed was defined by presence of skin bruising and/or petechiae in the lower extremities. Mucosal bleeds included epistaxis, gingival bleed, hematemesis, melena, hematochezia, menorrhagia, or hematuria (>5 red blood cells per high powered field) in a male or non-menstruating female. Any bleeding was defined by the presence of a skin bleed and/or mucosal bleed.

### Data analysis

Demographic and clinical features of CHIKV RT-PCR positive cases (i.e., only laboratory-confirmed chikungunya cases) were compared with two groups: all other AFI cases and DENV RT-PCR positive cases (i.e., laboratory-confirmed dengue cases). Cases that were only anti-CHIKV MAC-ELISA positive and CHIKV coinfections were not included in our analysis as laboratory-confirmed chikungunya cases. All other AFI cases included 6,916 laboratory-positive and laboratory-negative AFI cases that were not CHIKV RT-PCR positive (n = 1,499), anti-CHIKV MAC-ELISA positive (n = 136), anti-DENV MAC-ELISA positive (n = 285), dengue indeterminate (n = 51), or co-infected (n = 109) AFI cases. A dengue indeterminate case had a negative acute specimen and no serum collected ≥6 DPO available for testing. The number of laboratory-confirmed chikungunya, dengue and other AFI cases were plotted by month and year of illness onset and timing of presentation. Differences in proportions were tested by applying the Chi-square test, and medians were compared using the Mann-Whitney-Wilcoxon test. Bonferroni correction was used to account for simultaneous multiple comparisons. Multiple imputation was used to predict an independent plausible value for missing values (percent missing ranged from 0.4–8.6%) using generalized linear regression on non-missing variables to create 40 imputed complete data sets [41]. To identify predictors of chikungunya as compared to all other AFI cases and dengue cases separately, stepwise Akaike Information Criterion (AIC) variable selection was used for imputed datasets. Variables retained at least once in the 40 models were included in a pooled Poisson regression model (using weights to account for the pooling) before going through final variable selection [42]. From the final pooled Poisson regression model, relative risk and 95% confidence intervals (CI) were calculated for significant overall (all DPOs), early (<3 DPO), and late (3–5 DPO) predictors, along with any significant interactions with age group (<5-year-old, 5–19-year-old, 20–49-year-old, and ≥50-year-old). The final models included the age and the variables listed in S2 Table. The adjusted relative risks (aRR) were calculated by comparing cases with the significant predictor who were CHIKV RT-PCR positive to other AFI cases (or DENV RT-PCR positive), divided by the comparison of cases without the significant predictor who were CHIKV RT-PCR positive to other AFI cases (or DENV RT-PCR positive), and adjusted for the other statistically significant predictors in the models. Data were analyzed using the “mi” and “MASS” packages from R software (V3.3.0, R Foundation for Statistical Computing, Vienna, Austria).

## Results

Of the 8,996 participants enrolled in the AFI study, slightly more than half (54.8%, 4,930) had a pathogen detected, and 1,635 (18.2%) had a CHIKV infection [29]. In addition, 27 of the 109 participants with co-infections identified by molecular detection of two pathogens had a CHIKV infection. Most (91.7%, 1,499) of the chikungunya cases were confirmed by RT-PCR and were included in this analysis.

**Fig 1:**
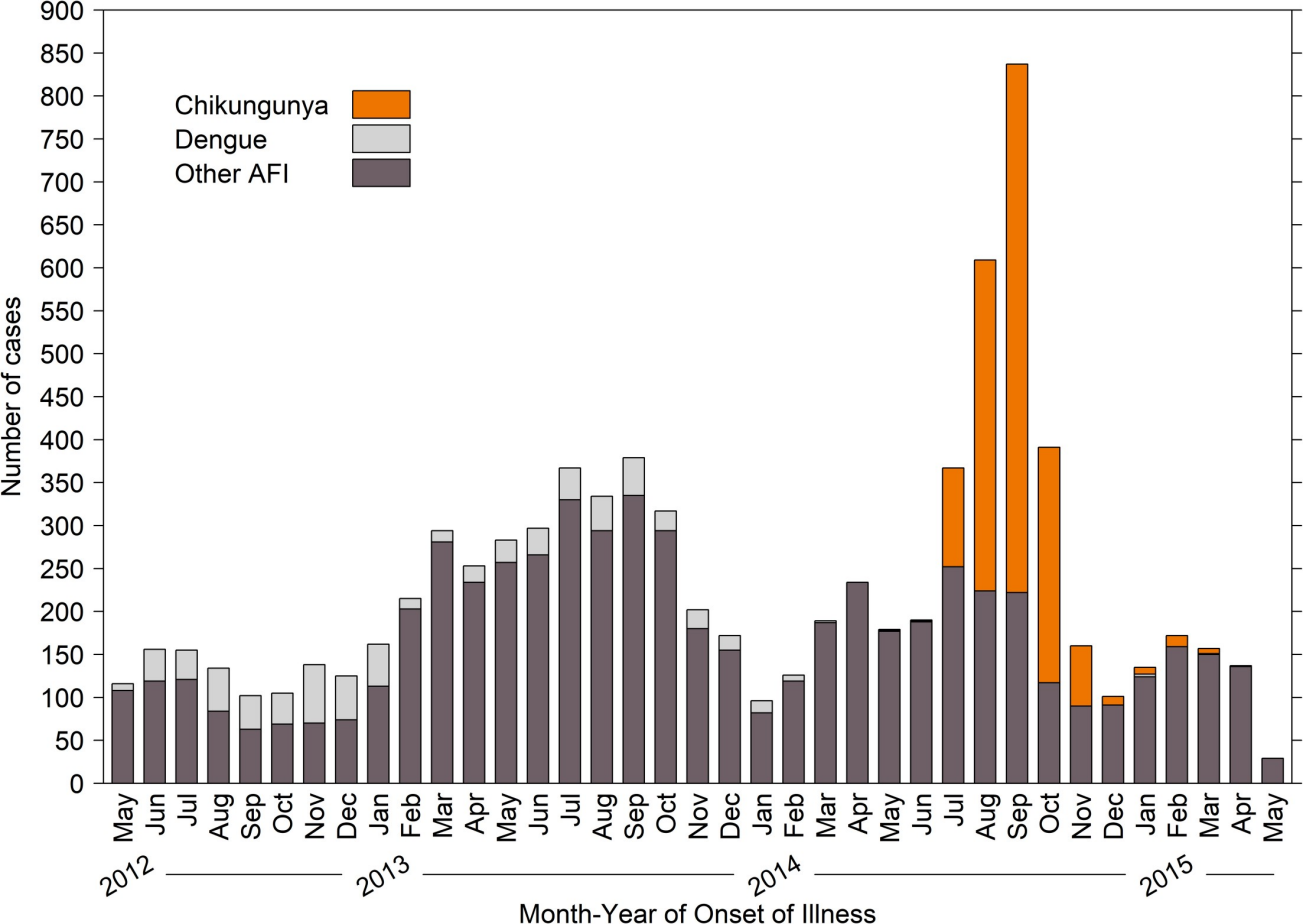
Number of acute febrile illness cases identified by month and year and pathogen detected, Acute Febrile Illness Study, May 7, 2012 –May 6, 2015, Puerto Rico.

The first chikungunya case was detected in May of 2014 and was followed by a six-month outbreak during which 1,574 cases were detected in 2014 (Fig 1). Only 61 chikungunya cases were detected in 2015. In contrast, most dengue cases were detected during a dengue outbreak that occurred in 2012 and continued through 2013, when a total of 921 dengue cases were detected. Few (n = 49) dengue cases were detected in 2014 to the end of the study in 2015. For this study, the 685 DENV RT-PCR positive cases were made up of 645 (94.2%) DENV-1, 38 (5.5%) DENV-4, and two (0.3%) DENV-2. The proportion of serotypes detected was consistent with what was in circulation throughout the island at the time [29].

### Participant demographics at enrollment between chikungunya cases, all other AFI cases, and dengue cases

A higher proportion (52.6%) of participants with chikungunya were female when compared with participants with dengue (45.3%, *P* = 0.002), as well as when compared to other AFI, although not statistically significant (49.8%, *P* = 0.052) (Table 1). Chikungunya cases were older on average than other AFI and dengue cases (median age of 24.8 years vs. 10.2, *P* <0.001; and 15.3 years, *P* <0.001, respectively), and likely because of this, a higher proportion of chikungunya cases reported having at least one chronic medical condition (39.5% vs. 32.7%, *P* <0.001; and 28.9%, *P* <0.001, respectively) (Table 1).

**Table 1:** Characteristics and clinical features of participants at study enrollment by diagnostic group, Acute Febrile Illness Study, May 7, 2012–May 6, 2015, Puerto Rico.

Parameters	Chikungunya N = 1,499		All Other AFI N = 6,916		P-value*	Dengue N = 685		P-value**
	N	%	N	%		N	%	
**Female, no. (%)**	788	52.6	3441	49.8	0.052	310	45.3	0.002
**Has chronic medical condition**	592	39.5	2261	32.7	<0.001	198	28.9	<0.001
**Median age, (range)**	24.8	(0.0–97.3)	10.2	(0.0–103.3)	<0.001	15.3	(0.0–77.5)	<0.001
**Age group, no. (%)**								
< 5 years old	219	14.6	2383	34.5	<0.001	51	7.4	<0.001
5–19 years old	420	28.0	2287	33.1	<0.001	433	63.2	<0.001
20–49 years old	474	31.6	1469	21.2	<0.001	146	21.3	<0.001
50 + years old	386	25.8	777	11.2	<0.001	55	8.0	<0.001
**Median DPO, (range)**	1	(0.0–6.0)	1	(0.0–8.0)	<0.001	3	(0.0–7.0)	<0.001
**Days post-illness onset, no. (%)**								
<3 days	1326	88.5	4892	70.7	<0.001	281	41.0	<0.001
3–5 days	161	10.7	1776	25.7	<0.001	382	55.8	<0.001
6–8 days	12	0.8	248	3.6	<0.001	22	3.2	<0.001
**Disposition, no. (%)**								
Admitted	161	10.7	1853	26.8	<0.001	306	44.7	<0.001
Died	2	0.1	13	0.2	0.908	1	0.1	1.000
Sent home	1335	89.1	5033	72.8	<0.001	376	54.9	<0.001
Transferred to other hospital	1	0.1	17	0.2	0.293	2	0.3	0.486

*P-value for the difference in proportion or median between RT-PCR-positive chikungunya and other AFI cases using the Chi-square test or Mann-Whitney Wilcoxon test, respectively.

**P-value for the difference in proportion or median between RT-PCR-positive chikungunya and RT-PCR-positive dengue cases using the Chi-square test or Mann-Whitney Wilcoxon test, respectively.

**Fig 2:**
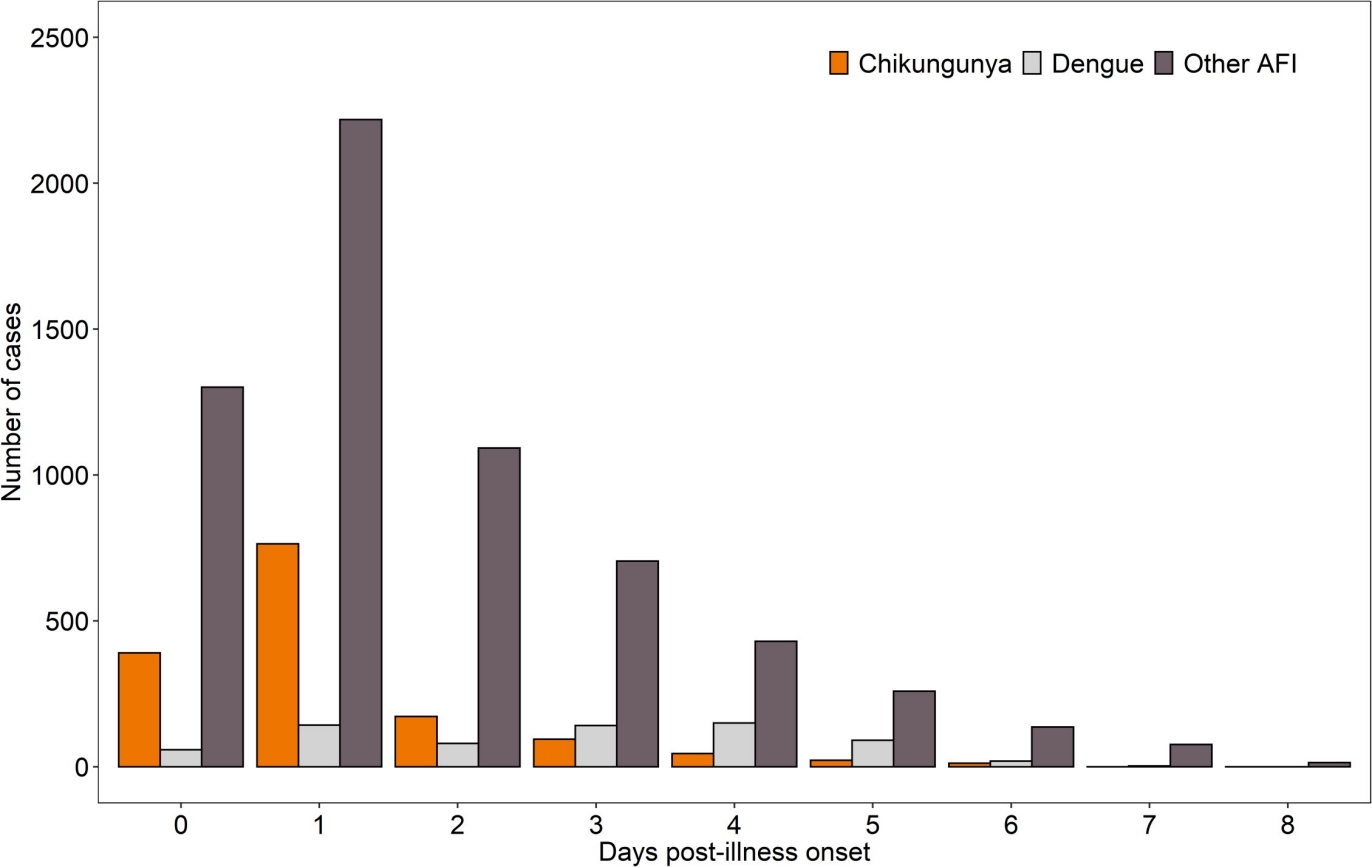
Timing of presentation for laboratory-confirmed chikungunya cases versus all other acute febrile illness cases and dengue cases.

The timing of initial presentation and disposition varied by comparator group (Table 1 and Fig 2). Chikungunya cases were more likely than other AFI cases to present early (<3 DPO) in the clinical course (88.5% vs. 70.7%, *P* <0.001); this difference was especially striking between chikungunya and dengue cases (88.5% vs. 41.0%, *P* <0.001). Chikungunya cases were less likely than other AFI cases to be admitted to the hospital at enrollment (10.7% vs. 26.8%, *P* <0.001), and again, this difference was more pronounced between chikungunya and dengue cases (10.7% vs. 44.7%, *P* <0.001).

### Comparison of signs and symptoms by group

A significantly (at *P* <0.001) higher proportion of chikungunya cases than other AFI cases had muscle, bone or back pain (85.7% vs. 53.6%, respectively), joint pain (82.0% vs. 41.0%), headache (71.7% vs. 62.1%), skin rash (61.2% vs. 20.4%), red conjunctiva (57.9% vs. 47.5%), facial and/or neck erythema (57.0% vs 35.2%), any bleeding (48.0% vs. 24.4%), skin bleeding (39.0% vs. 11.2%), red swollen joints (43.3% vs. 9.3%), and pruritic skin (30.0% vs. 12.4%) at study enrollment (S1 Table). These significant differences were sustained (except for red conjunctiva, and facial and/or neck erythema), although not as pronounced, when the comparison was made between chikungunya and dengue cases. In contrast, a significantly (at *P* <0.001) higher proportion of dengue than chikungunya cases had headache (83.4% vs. 71.7%), chills (78.4% vs. 71.0%), anorexia (77.1% vs. 56.4%), dizziness (61.2% vs. 43.1%), eye pain (56.8% vs. 46.4%), gastro-intestinal symptoms, such as: nausea (66.7% vs. 42.9%), abdominal pain (55.6% vs. 31.2%), diarrhea (34.9% vs. 17.3%) and vomiting (25.1% vs. 13.9%), and signs of poor circulation (48.0% vs. 31.8%) at study enrollment. Chikungunya cases were significantly less likely than other AFI cases and dengue cases to have respiratory tract symptoms, such as cough (25.2% vs. 60.1% and 35.2% respectively), and sore throat (21.0% vs. 42.5% and 31.2% respectively). Chikungunya cases were significantly less likely than other AFI cases to be hemoconcentrated or have thrombocytopenia, but there was no difference in the proportion with leukopenia. A higher proportion of dengue than chikungunya cases had moderate hemoconcentration (3.1% vs. 0.8%, *P* <0.001), severe hemoconcentration (1.0% vs. 0.2%, *P* = 0.022), thrombocytopenia (35.2% vs. 2.0%, *P* <0.001), and leukopenia (75.9% vs. 19.9%, *P* <0.001).

### Overall predictors of laboratory-confirmed chikungunya regardless of DPO

In multivariate analyses, when we compared 1,499 chikungunya cases to 6,916 other AFI cases regardless of DPO at presentation, the positive predictors of chikungunya among participants of all ages were joint pain, muscle, bone or back pain, red conjunctiva, and skin rash, although the strength of skin rash as a predictor varied significantly across age groups (Tables 2 and 3). Thrombocytopenia, cough, sore throat, rhinorrhea, signs of poor circulation, gastrointestinal symptoms such as diarrhea and abdominal pain, headache, and anorexia were negative predictors of chikungunya among participants of all ages regardless of DPO. When we compared 1,499 chikungunya cases and 685 dengue cases regardless of DPO, we found that red swollen joints, joint pain, skin rash, any bleeding, and irritability were significant positive predictors of chikungunya among participants of all ages (Tables 2 and 3). Thrombocytopenia, nausea, signs of poor circulation, cough, headache, diarrhea, eye pain, dizziness, and leukopenia were independent negative predictors for chikungunya when compared with dengue cases although the strength of leukopenia as a predictor varied significantly across age groups (Table 3).

**Table 2:** Predictors of laboratory-confirmed chikungunya versus all other acute febrile illnesses or laboratory-confirmed dengue for all ages regardless of the timing of presentation, Acute Febrile Illness Study, May 7, 2012–May 6, 2015, Puerto Rico.

Predictors	Chikungunya N = 1,499		All Other AFI N = 6,916		aRR* (95% CI)	Dengue N = 685		aRR** (95% CI)
	N	%	N	%		N	%	
**Positive Predictors**								
Red swollen joints	649	43.3	-	-	-	81	11.8	4.53 (3.08–6.66)
Joint pain	1229	82.0	2834	41.0	2.30 (1.86–2.84)	389	56.8	3.30 (2.29–4.76)
Skin rash	917	61.2	-	-	-	301	43.9	2.79 (1.99–3.92)
Any bleeding*	720	48.0	-	-	-	247	36.1	1.74 (1.25–2.42)
Irritability	448	29.9	-	-	-	184	26.9	1.63 (1.16–2.30)
Muscle/bone/back pain	1284	85.7	3708	53.6	1.40 (1.10–1.78)	-	-	-
Red conjunctiva	868	57.9	3285	47.5	1.14 (1.02–1.28)	-	-	-
**Negative Predictors**										
Thrombocytopenia	30	2.0	422	6.1	0.35 (0.24–0.51)	241	35.2	0.08 (0.04–0.17)
Nausea	643	42.9	-	-	-	457	66.7	0.42 (0.31–0.59)
Cough	377	25.2	4155	60.1	0.56 (0.49–0.64)	241	35.2	0.57 (0.41–0.77)
Sore throat	315	21.0	2939	42.5	0.67 (0.58–0.77)	-	-	-
Rhinorrhea	397	26.5	3805	55.0	0.71 (0.63–0.82)	-	-	-
Signs of poor circulation^†^	476	31.8	2645	38.2	0.79 (0.69–0.91)	329	48.0	0.54 (0.39–0.75)
Diarrhea	260	17.3	1879	27.2	0.79 (0.69–0.91)	239	34.9	0.64 (0.45–0.92)
Abdominal pain	467	31.2	2924	42.3	0.80 (0.70–0.90)	-	-	-
Headache	1075	71.7	4297	62.1	0.84 (0.74–0.97)	571	83.4	0.61 (0.40–0.91)
Eye pain	695	46.4	-	-	-	389	56.8	0.65 (0.47–0.89)
Anorexia	845	56.4	4583	66.3	0.86 (0.77–0.96)	-	-	-
Dizziness	646	43.1	-	-	-	419	61.2	0.67 (0.48–0.95)

*Any bleeding was defined by the presence of a skin bleed and/or mucosal bleed.

**Adjusted relative risk (aRR) based on pooled Poisson regression model.

(-) Indicates no significant predictor between chikungunya and comparator group.

† Signs of poor circulation included report of pale cold skin and/or having cyanosis.

**Table 3:** Age group-specific predictors of laboratory-confirmed chikungunya versus all other acute febrile illnesses or laboratory-confirmed dengue regardless of the timing of presentation, Acute Febrile Illness Study, May 7, 2012–May 6, 2015, Puerto Rico.

Predictors	<5 years old (219 CHIKV vs. 2,602 AFI) aRR (95% CI)	5–19 years old (420 CHIKV vs. 2,707 AFI) aRR (95% CI)	20–49 years old (474 CHIKV vs. 943 AFI) aRR (95% CI)	50+ years old (386 CHIKV vs. 1,163 AFI) aRR (95% CI)
**Chikungunya vs. AFI**				
Skin rash*	**3.82**^†^ **(2.72–5.37)**	**3.93**^†^ **(3.00–5.14)**	**2.58**^†^ **(2.05–3.24)**	**1.38**^†^ **(1.09–1.76)**
Red swollen joints	1.08 (0.77–1.53)	**1.27**^†^ **(1.03–1.58)**	**1.86**^†^ **(1.52–2.27)**	**1.98**^†^ **(1.59–2.46)**
Facial and/or neck erythema	**1.70**^†^ **(1.24–2.31)**	1.14 (0.90–1.46)	0.88 (0.71–1.08)	1.10 (0.87–1.38)
Any bleeding	**1.63**^†^ **(1.21–2.18)**	1.17 (0.95–1.44)	1.01 (0.83–1.22)	1.18 (0.96–1.46)
Leukopenia	**0.51**^¥^ **(0.30–0.88)**	**0.63**^¥^ **(0.49–0.81)**	1.05 (0.85–1.30)	1.20 (0.94–1.52)
**Chikungunya vs. Dengue**				
Leukopenia	**0.08**^¥^ **(0.03–0.19)**	**0.07**^¥^ **(0.04–0.11)**	**0.13**^¥^ **(0.08–0.2)**	**0.27**^¥^ **(0.13–0.57)**

* Age significantly affects predictor such that the adjusted relative risk (aRR) magnitude is significantly different across age groups.

† Significant positive predictor

¥ Significant negative predictor

In the chikungunya versus all other AFI comparison, some clinical features were only statistically significant when interacted by age group (Table 3). Having red swollen joints was a positive predictor of chikungunya when compared with all other AFI cases among participants >5 years old. Face and/or neck erythema, and any bleeding were positive predictors of chikungunya only among participants <5 years old. Leukopenia was a negative predictor of chikungunya among participants <20 years old.

### Early predictors of laboratory-confirmed chikungunya

When we compared 1,326 chikungunya cases to 4,892 other AFI cases that presented early (<3 DPO), the significant early positive predictors of chikungunya among participants of all ages in multivariate analyses were joint pain, muscle, bone or back pain, and skin rash, although the strength of skin rash as a predictor varied significantly across age groups (Tables 4 and 5). Thrombocytopenia, respiratory symptoms (i.e., cough, sore throat and rhinorrhea), signs of poor circulation, gastrointestinal symptoms (i.e., abdominal pain, diarrhea and nausea), and anorexia were early negative predictors of chikungunya when compared to other AFI cases. When compared with the 281 dengue cases that presented <3 DPO, joint pain and red swollen joints were significant early positive predictors of chikungunya in all age groups (Table 4). In contrast to the chikungunya vs. all other AFI comparison, skin rash was an early positive predictor (aRR = 1.55, 95% CI = 1.22–1.97) only among participants 5-19-years-old in the chikungunya vs. dengue comparison (Table 5). Thrombocytopenia, leukopenia and nausea were significant early negative predictors of chikungunya when compared to dengue cases (Table 4).

**Table 4:** Early predictors of laboratory-confirmed chikungunya versus all other acute febrile illnesses or laboratory-confirmed dengue for participants of all ages, Acute Febrile Illness Study, May 7, 2012–May 6, 2015, Puerto Rico.

Predictors	Chikungunya N = 1,326		All Other AFI N = 4,892		aRR* (95% CI)	Dengue N = 281		aRR* (95% CI)
	N	%	N	%		N	%	
**Positive Predictors**								
Joint pain	1088	85.1	1844	37.7	2.26 (1.79–2.85)	140	49.8	1.26 (1.06–1.50)
Muscle, bone or back pain	1133	85.4	2422	49.5	1.35 (1.05–1.75)	-	-	-
Red swollen joints	565	42.6	-	-	-	20	7.1	1.13 (1.01–1.28)
**Negative Predictors**								
Thrombocytopenia	23	1.7	131	2.7	0.53 (0.36–0.79)	42	14.9	0.60 (0.38–0.93)
Cough	331	25.0	2842	58.1	0.60 (0.52–0.69)	-	-	-
Sore throat	279	21.0	1969	40.2	0.71 (0.61–0.82)	-	-	-
Rhinorrhea	351	26.5	2661	54.4	0.72 (0.63–0.83)	-	-	-
Leukopenia	221	16.7	-	-	-	166	59.1	0.73 (0.63–0.84)
Signs of poor circulation^†^	405	30.5	1688	34.5	0.82 (0.72–0.92)	-	-	-
Abdominal pain	403	30.4	1927	39.4	0.84 (0.74–0.97)	-	-	-
Diarrhea	207	15.6	1133	23.2	0.85 (0.73–0.99)	-	-	-
Nausea	549	41.4	2299	47.0	0.87 (0.77–0.99)	165	58.7	0.88 (0.78–0.98)
Anorexia	725	54.7	3088	63.1	0.88 (0.78–0.99)	-	-	-

*Adjusted relative risk (aRR) based on pooled Poisson regression model.

(-) Indicates no significant predictor between chikungunya and comparator group.

† Signs of poor circulation included report of pale cold skin and/or having cyanosis.

**Table 5:** Age group-specific early predictors of laboratory-confirmed chikungunya versus all other acute febrile illnesses or laboratory-confirmed dengue, Acute Febrile Illness Study, May 7, 2012–May 6, 2015, Puerto Rico.

Predictors	<5 years old (204 CHIKV vs. 1872 AFI) aRR (95% CI)	5–19 years old (387 CHIKV vs. 1587 AFI) aRR (95% CI)	20–49 years old (411 CHIKV vs. 962 AFI) aRR (95% CI)	50+ years old (324 CHIKV vs. 471 AFI) aRR (95% CI)
**Chikungunya vs. AFI**				
Skin rash	**3.96 (2.77–5.68)**	**4.03 (3.04–5.35)**	**2.44 (1.91–3.11)**	**1.36 (1.04–1.77)**
Red swollen joints	1.18 (0.82–1.68)	1.20 (0.95–1.50)	**1.76 (1.42–2.19)**	**1.83 (1.44–2.32)**
Any bleeding	**1.69 (1.24–2.29)**	1.15 (0.93–1.44)	1.01 (0.82–1.24)	1.13 (0.90–1.43)
Face or neck erythema	1.18 (0.82–1.68)	1.20 (0.95–1.50)	**1.76 (1.42–2.19)**	**1.83 (1.44–2.32)**
**Chikungunya vs. Dengue**				
Skin rash	1.03 (0.75–1.42)	**1.55 (1.22–1.97)**	1.10 (0.90–1.35)	1.03 (0.82–1.30)

Some clinical features were significant predictors of chikungunya compared to all other AFI cases when interacted with age group (Table 5). Report of any bleeding was an independent, positive early predictor of chikungunya among participants <5 years old. Having red swollen joints was a positive predictor of chikungunya among participants >20 years old. Facial and/or neck erythema was a positive predictor among participants >20-years old.

### Late predictors of laboratory-confirmed chikungunya

When we compared 161 chikungunya cases to 1,776 other AFI cases that presented 3–5 DPO, the independent significant positive predictors of chikungunya among participants of all ages were red swollen joints, skin rash, pruritic skin, and red conjunctiva (Table 6). Absence of thrombocytopenia, sore throat, rhinorrhea, and signs of poor circulation were significant negative predictors of chikungunya. When the 382 dengue cases were the comparator group, red swollen joints and pruritic skin were independent significant positive predictors of chikungunya at 3–5 DPO across all age groups (Table 6). Absence of thrombocytopenia, leukopenia, nausea, and cough were negative predictors of chikungunya. Age did not significantly affect any predictor among those presenting 3–5 DPO.

**Table 6:** Late predictors of laboratory-confirmed chikungunya versus all other acute febrile illnesses or laboratory-confirmed dengue for participants of all ages, Acute Febrile Illness Study, May 7, 2012–May 6, 2015, Puerto Rico.

Predictors	Chikungunya N = 161		All Other AFI N = 1,776		aRR* (95% CI)	Dengue N = 382		aRR* (95% CI)
	N	%	N	%		N	%	
**Positive Predictors**								
Red swollen joints	76	47.2	182	10.2	2.69 (1.89–3.81)	55	14.4	1.82 (1.31–2.53)
Skin rash	98	60.9	475	26.7	2.40 (1.65–3.50)	-	-	-
Pruritic skin	80	49.7	290	16.3	1.69 (1.16–2.46)	95	24.9	1.74 (1.25–2.42)
Red conjunctiva	98	60.9	896	50.5	1.44 (1.03–2.02)	-	-	-
**Negative Predictors**								
Thrombocytopenia	7	4.3	237	13.3	0.22 (0.11–0.46)	185	48.4	0.17 (0.08–0.36)
Leukopenia	69	42.9	-	-	-	335	87.7	0.38 (0.27–0.52)
Sore throat	34	21.1	841	47.4	0.44 (0.29–0.65)	-	-	-
Rhinorrhea	44	27.3	986	55.5	0.52 (0.35–0.75)	-	-	-
Sign of poor circulation^†^	65	40.4	835	47.0	0.64 (0.46–0.89)	-	-	-
Nausea	88	54.7	-	-	-	277	72.5	0.65 (0.47–0.91)
Cough	45	28.0	-	-	-	153	40.1	0.67 (0.47–0.96)

* Adjusted relative risk (aRR) based on pooled Poisson regression model.

(-) Indicates no significant predictor between chikungunya and comparator group.

† Signs of poor circulation included report of pale cold skin and/or having cyanosis.

## Discussion

Of the 8,996 participants enrolled in our AFI study, nearly one-fifth had chikungunya. Chikungunya cases were more likely than other AFI cases to be older and a higher proportion reported having at least one chronic medical condition. This pattern of disease has been seen in other areas with recent CHIKV emergence [43, 44] and may be due to differences in health-seeking behaviors and/or complications among older individuals with preexisting co-morbidities, including osteoarthritis. As has been previously reported, chikungunya cases were more likely than other AFI cases to present early in the clinical course [45–49]; differences that were especially pronounced between chikungunya and dengue cases [50–52]. Whether the difference in the timing of presentation is due to a more abrupt onset of fever and the occurrence of very high fever (≥40°C) among those with chikungunya than dengue remains unknown. In our study, we did not collect information about the degree of fever or the fever curve to be able to confirm these findings.

As a clinical syndrome, AFIs are a diagnostic challenge for clinicians especially early in the clinical course when anticipatory guidance and supportive care may pre-empt medical complications. In our study, we identified clinical predictors for RT-PCR-positive chikungunya cases by timing of presentation and patient age using two clinical comparators. While there are a few recent prospective studies that sought to identify predictors of chikungunya using dengue cases [43, 44, 48, 53–55] or all other AFIs [43, 55] as the clinical comparator, many were biased by restrictive study inclusion criteria [44, 48, 53–55] including the use of dengue and/or chikungunya case definition [44, 53]. In addition, some studies included only hospitalized cases [44, 46, 50], or restricted the study to adult [44, 50, 55] or pediatric cases [46, 48]. Lastly, many of these studies were limited by small sample size (i.e., <50 chikungunya cases) [43, 44, 48, 53, 55], and because of this, other investigators found few or no clinical features that distinguished chikungunya cases from other AFIs [44, 53, 55].

We identified seven predictors of laboratory-confirmed chikungunya among AFI patients regardless of the timing of presentation or comparison group used including two positive predictors, joint pain and skin rash, and five negative predictors: thrombocytopenia, signs of poor circulation, headache, cough, and diarrhea. Red swollen joints was also a predictor, except for patients aged <5 years when compared to all other AFI. Similarly, leukopenia was a negative predictor, except in adults when compared to all other AFI and in all age groups when compared to dengue. Last, classic signs and symptoms of dengue including headache, eye pain, and signs of poor circulation were negative predictors of chikungunya when compared with dengue.

While more recent studies have not found bleeding to be an early predictor of chikungunya, many of the original chikungunya case reports described bleeding among chikungunya cases including epistaxis and petechiae [46, 56, 57]. In our study, a significantly higher proportion of chikungunya cases had skin bleeding (mostly petechiae) when compared to dengue cases, and because of this, any bleeding was a significant positive predictor of chikungunya when compared with dengue for all ages regardless of the timing of presentation. Any bleeding was also a significant positive predictor of chikungunya overall and early in the clinical course among participants <5 years when compared with all other AFI cases. A study by Velasco et al. also identified differences in signs and symptoms by the age of the chikungunya patient in that children (<18 years) were more likely to have rash while those ≥18 years were more likely to have bleeding [53]. In our study, this finding was mainly due to differences in the occurrence of skin bleeding, and specifically, petechiae on the lower extremities (44.3% or 97 of 219 chikungunya cases < 5 years had petechiae vs. 8.5% or 203 of 2,383 of all other AFI cases < 5 years old, (P <0.001). While skin bleeding, particularly petechiae, may be challenging to correctly identify especially in patients with darkly pigmented skin, young children with chikungunya were probably more likely to present with petechiae due to the higher incidence of minor lower extremity trauma in this age group. Nevertheless, our study explored petechiae in the lower extremities only and may have missed petechial skin bleeding caused by other AFI that present on the face and upper trunk of young children because of frequent and/or severe cough spells or vomits [58]. In addition, viral genomic studies have identified respiratory viruses, such as the respiratory syncytial virus, rhinovirus, and influenza in nasopharyngeal aspirates of young children with signs and symptoms of respiratory infection and petechiae [59].

Making a clinical diagnosis early in the clinical course is difficult but is important to guide patient management and administer anticipatory guidance for timely follow-up. We found that for AFI patients of all ages presenting early (<3 DPO) in the clinical course, there were three predictors of chikungunya regardless of the comparison group used. These early predictors included joint pain and the absence of thrombocytopenia and nausea. In addition, having a red swollen joint was a positive predictor in all age groups if dengue was used as a comparator, and in participants ≥20 years old when all other AFIs was the comparator. In contrast, skin rash was a positive predictor in all age groups if all other AFI was used as a comparator and only in the 5-19-year-old group if dengue was the comparator. While skin rash as a predictor was somewhat surprising, several similar, albeit smaller, studies found early skin rash to be predictive of chikungunya when compared with dengue cases [43, 46, 48]. Not surprisingly, the absence of respiratory (cough, sore throat, rhinorrhea) or gastrointestinal symptoms (abdominal pain, diarrhea, anorexia) or signs of poor circulation predicted chikungunya at <3 DPO if all other AFIs was the clinical comparator. Not being leukopenic and absence of nausea were predictive of chikungunya if dengue was the clinical comparator.

In general, late (3–5 DPO) predictors of chikungunya were more specific than early predictors. For example, joint, muscle, bone or back pain were no longer predictive of chikungunya while arthritis became predictive in all age-groups, and having pruritic skin became a predictor regardless of the comparison group used. While the absence of thrombocytopenia was a significant predictor of chikungunya regardless of the comparison group used, leukopenia was only a predictor when dengue was the comparator group. Our late chikungunya predictor findings were as expected in that dengue cases are more likely to be leukopenic, thrombocytopenic, and have signs of vascular leakage including nausea and cough at 3–5 DPO [29, 46]; gastrointestinal and respiratory symptoms are uncommon among those with chikungunya [50].

While our study had more laboratory-confirmed chikungunya cases than other prospective studies and enrolled all patients presenting with fever regardless of age or presenting clinical characteristics, it may be limited in generalizability as previously addressed [29]. Second, our analysis of predictors utilizing dengue as a comparator group may be limited because chikungunya cases presented earlier in the clinical course than dengue cases. However, we still had more, late-presenting chikungunya cases than most studies had cases in total. Last, chikungunya and dengue cases presented at different time periods during the study during two separate outbreaks as described earlier. Due to the increased public awareness of chikungunya during the 2014–2015 outbreak, patients at enrollment (i.e., before the laboratory diagnosis) may have been more likely to report joint pain and perhaps, muscle, bone and back pain than during the 2012–2013 dengue outbreak. This may have biased the reporting of these symptoms.

Our findings demonstrate that chikungunya does have signs and symptoms that distinguish it from other AFIs and dengue regardless of timing of presentation and age of patient. Clinicians can use these findings to identify cases of chikungunya and rule out cases so that other AFIs that require timely anticipatory guidance and clinical management can be identified. While our previous study suggested that the presence of leukopenia and thrombocytopenia were the best predictors of dengue, chikungunya does not require that a complete blood count be done.

### Data availability statement

All relevant data are within the paper and its Supporting Information files.

## Supporting information

S1 Checklist STROBE statement. (DOCX)

S1 Table Clinical features of participants at study enrollment by diagnostic group, Acute Febrile Illness Study, May 7, 2012–May 6, 2015, Puerto Rico. (DOCX)

S2 Table Predictors of laboratory-confirmed chikungunya versus all other acute febrile illnesses (AFI) and laboratory-confirmed dengue by timing of presentation. (DOCX)
