# Supplementary material for: Distinguishing patients with laboratory-confirmed chikungunya from dengue and other acute febrile illnesses, Puerto Rico, 2012–2015
Source: PLoS Negl Trop Dis. 2019 Jul 22;13(7):e0007562. doi: 10.1371/journal.pntd.0007562 (PMC6645456; doi:10.1371/journal.pntd.0007562)
Supplement: S1 Checklist — (DOCX) [file pntd.0007562.s001.docx]

**[S1](http://journals.plos.org/plosntds/article/asset?unique&id=info:doi/10.1371/journal.pntd.0005859.s001)** [**Checklist.**](http://journals.plos.org/plosntds/article/asset?unique&id=info:doi/10.1371/journal.pntd.0005859.s001) **STROBE statement.**

STROBE Statement—checklist of items that should be included in reports of observational studies

|  | Item No. | Recommendation | Page  No. | Relevant text from manuscript |
| --- | --- | --- | --- | --- |
| **Title and abstract** | 1 | (*a*) Indicate the study’s design with a commonly used term in the title or the abstract | 1 | Prospective study of Acute Febrile Illnesses |
|  |  | (*b*) Provide in the abstract an informative and balanced summary of what was done and what was found | 3-4 | Lines 45-71 |
| Introduction | | | |  |
| Background/rationale | 2 | Explain the scientific background and rationale for the investigation being reported | 6-8 | Lines 92-150 |
| Objectives | 3 | State specific objectives, including any prespecified hypotheses | 8 | Lines 145-147 |
| Methods | | | |  |
| Study design | 4 | Present key elements of study design early in the paper | 8 | Lines 147-150 |
| Setting | 5 | Describe the setting, locations, and relevant dates, including periods of recruitment, exposure, follow-up, and data collection | 9-10 | Lines 164-203 |
| Participants | 6 | (*a*) *Cohort study*—Give the eligibility criteria, and the sources and methods of selection of participants. Describe methods of follow-up  *Case-control study*—Give the eligibility criteria, and the sources and methods of case ascertainment and control selection. Give the rationale for the choice of cases and controls  *Cross-sectional study*—Give the eligibility criteria, and the sources and methods of selection of participants | 9 | Lines 172-180 |
|  |  | (*b*) *Cohort study*—For matched studies, give matching criteria and number of exposed and unexposed  *Case-control study*—For matched studies, give matching criteria and the number of controls per case | NA | NA, no matching |
| Variables | 7 | Clearly define all outcomes, exposures, predictors, potential confounders, and effect modifiers. Give diagnostic criteria, if applicable | 11 | Lines 204-223 |
| Data sources/ measurement | 8* | For each variable of interest, give sources of data and details of methods of assessment (measurement). Describe comparability of assessment methods if there is more than one group | 9-10 | Lines 175-178  Lines 181-203 |
| Bias | 9 | Describe any efforts to address potential sources of bias | 11-12 | Lines 214-223 |
| Study size | 10 | Explain how the study size was arrived at | NA | NA |

Continued on next page

| Quantitative variables | 11 | Explain how quantitative variables were handled in the analyses. If applicable, describe which groupings were chosen and why | 11-12 | Lines 214-239 |
| --- | --- | --- | --- | --- |
| Statistical methods | 12 | (*a*) Describe all statistical methods, including those used to control for confounding | 11-12 | Lines 225-243 |
|  |  | (*b*) Describe any methods used to examine subgroups and interactions | 12 | Lines 230-244 |
|  |  | (*c*) Explain how missing data were addressed | 12 | Lines 227-230 |
|  |  | (*d*) *Cohort study*—If applicable, explain how loss to follow-up was addressed  *Case-control study*—If applicable, explain how matching of cases and controls was addressed  *Cross-sectional study*—If applicable, describe analytical methods taking account of sampling strategy | NA | NA |
|  |  | (*e*) Describe any sensitivity analyses | NA | NA |
| Results | | | | |
| Participants | 13* | (a) Report numbers of individuals at each stage of study—eg numbers potentially eligible, examined for eligibility, confirmed eligible, included in the study, completing follow-up, and analysed | 13 | Lines 249-260 |
|  |  | (b) Give reasons for non-participation at each stage | 13 | Lines 249-260 |
|  |  | (c) Consider use of a flow diagram |  |  |
| Descriptive data | 14* | (a) Give characteristics of study participants (eg demographic, clinical, social) and information on exposures and potential confounders | 13-14  15-16  17  S2 Table | Lines 265-273  Table 1  Lines 274-281  Lines 282-288  Supplemental Table 1 |
|  |  | (b) Indicate number of participants with missing data for each variable of interest | NA | NA |
|  |  | (c) *Cohort study*—Summarise follow-up time (eg, average and total amount) | Figure 1  Figure 2 | Figure 1  Figure 2 |
| Outcome data | 15* | *Cohort study*—Report numbers of outcome events or summary measures over time | NA | NA |
|  |  | *Case-control study—*Report numbers in each exposure category, or summary measures of exposure | NA | NA |
|  |  | *Cross-sectional study—*Report numbers of outcome events or summary measures | NA | NA |
| Main results | 16 | (*a*) Give unadjusted estimates and, if applicable, confounder-adjusted estimates and their precision (eg, 95% confidence interval). Make clear which confounders were adjusted for and why they were included | 17-19  20-21  22  23  24  25  26  27 | Lines 293-338  Table 2  Lines 339-346  Table 3  Lines 347-354  Lines 355-376  Table 4  Lines 377-382  Table 5  Lines 383-385  Line 386-395  Table 6  Lines 396-401 |
|  |  | (*b*) Report category boundaries when continuous variables were categorized | 11 | Lines 204-212 |
|  |  | (*c*) If relevant, consider translating estimates of relative risk into absolute risk for a meaningful time period | NA | NA |

Continued on next page

| Other analyses | 17 | Report other analyses done—eg analyses of subgroups and interactions, and sensitivity analyses | NA | NA |
| --- | --- | --- | --- | --- |
| Discussion | | | | |
| Key results | 18 | Summarize key results with reference to study objectives | 28-31 | 403-488 |
| Limitations | 19 | Discuss limitations of the study, taking into account sources of potential bias or imprecision. Discuss both direction and magnitude of any potential bias | 31-32 | 490-501 |
| Interpretation | 20 | Give a cautious overall interpretation of results considering objectives, limitations, multiplicity of analyses, results from similar studies, and other relevant evidence | 32 | 503-505 |
| Generalizability | 21 | Discuss the generalizability (external validity) of the study results | 30 | 505-509 |
| Other information | |  | | |
| Funding | 22 | Give the source of funding and the role of the funders for the present study and, if applicable, for the original study on which the present article is based | In Editorial Manager | In Editorial Manager |

*Give information separately for cases and controls in case-control studies and, if applicable, for exposed and unexposed groups in cohort and cross-sectional studies.

**Note:** An Explanation and Elaboration article discusses each checklist item and gives methodological background and published examples of transparent reporting. The STROBE checklist is best used in conjunction with this article (freely available on the Web sites of PLoS Medicine at http://www.plosmedicine.org/, Annals of Internal Medicine at http://www.annals.org/, and Epidemiology at http://www.epidem.com/). Information on the STROBE Initiative is available at www.strobe-statement.org.
