# Supplementary material for: Distinguishing patients with laboratory-confirmed chikungunya from dengue and other acute febrile illnesses, Puerto Rico, 2012–2015
Source: PLoS Negl Trop Dis. 2019 Jul 22;13(7):e0007562. doi: 10.1371/journal.pntd.0007562 (PMC6645456; doi:10.1371/journal.pntd.0007562)
Supplement: S1 Table — (DOCX) [file pntd.0007562.s002.docx]

**S1 Table.** Clinical features of participants at study enrollment by diagnostic group, Acute Febrile Illness Study, May 7, 2012 ̶ May 6, 2015, Puerto Rico.

| **Clinical features of participants at study enrollment** | **Chikungunya** | | **All Other AFI** | | **P-value^*^** | **Dengue** | | **P-value^**^** |
| --- | --- | --- | --- | --- | --- | --- | --- | --- |
|  | **N=1,499** | | **N=6,916** | |  | **N=685** | |  |
|  | **N** | **%** | **N** | **%** |  | **N** | **%** |  |
| **Signs and symptoms, no. (%)** |  |  |  |  |  |  |  |  |
| Muscle/bone/back pain | 1284 | 85.7 | 3708 | 53.6 | <0.001 | 507 | 74.0 | <0.001 |
| Joint pain | 1229 | 82.0 | 2834 | 41.0 | <0.001 | 389 | 56.8 | <0.001 |
| Red/swollen joints | 649 | 43.3 | 643 | 9.3 | <0.001 | 81 | 11.8 | <0.001 |
| Headache | 1075 | 71.7 | 4297 | 62.1 | <0.001 | 571 | 83.4 | <0.001 |
| Red conjunctiva | 868 | 57.9 | 3285 | 47.5 | <0.001 | 375 | 54.7 | 0.181 |
| Eye pain | 695 | 46.4 | 2532 | 36.6 | <0.001 | 389 | 56.8 | <0.001 |
| Skin rash | 917 | 61.2 | 1411 | 20.4 | <0.001 | 301 | 43.9 | <0.001 |
| Facial and/or neck erythema | 855 | 57.0 | 2436 | 35.2 | <0.001 | 377 | 55.0 | 0.407 |
| Pruritic skin | 449 | 30.0 | 855 | 12.4 | <0.001 | 148 | 21.6 | <0.001 |
| Jaundice/icteric sclera | 23 | 1.5 | 156 | 2.3 | 0.098 | 24 | 3.5 | 0.005 |
| Any bleeding | 720 | 48.0 | 1685 | 24.4 | <0.001 | 247 | 36.1 | <0.001 |
| Skin bleeding | 585 | 39.0 | 777 | 11.2 | <0.001 | 165 | 24.1 | <0.001 |
| Mucosal bleeding | 256 | 17.1 | 1100 | 15.9 | 0.280 | 123 | 18.0 | 0.659 |
| Tiredness, lethargy | 1212 | 80.9 | 4928 | 71.3 | <0.001 | 587 | 85.7 | 0.007 |
| Nervousness, anxiety | 490 | 32.7 | 1966 | 28.4 | 0.001 | 210 | 30.7 | 0.371 |
| Irritability | 448 | 29.9 | 1993 | 28.8 | 0.426 | 184 | 26.9 | 0.163 |
| Chills | 1065 | 71.0 | 4323 | 62.5 | <0.001 | 537 | 78.4 | <0.001 |
| Sign of poor circulation^†^ | 476 | 31.8 | 2645 | 38.2 | <0.001 | 329 | 48.0 | <0.001 |
| Dizziness | 646 | 43.1 | 2460 | 35.6 | <0.001 | 419 | 61.2 | <0.001 |
| Rhinorrhea | 397 | 26.5 | 3805 | 55.0 | <0.001 | 171 | 25.0 | 0.485 |
| Cough | 377 | 25.2 | 4155 | 60.1 | <0.001 | 241 | 35.2 | <0.001 |
| Sore throat | 315 | 21.0 | 2939 | 42.5 | <0.001 | 214 | 31.2 | <0.001 |
| Anorexia | 845 | 56.4 | 4583 | 66.3 | <0.001 | 528 | 77.1 | <0.001 |
| Nausea | 643 | 42.9 | 3425 | 49.5 | <0.001 | 457 | 66.7 | <0.001 |
| Abdominal pain | 467 | 31.2 | 2924 | 42.3 | <0.001 | 381 | 55.6 | <0.001 |
| Diarrhea | 260 | 17.3 | 1879 | 27.2 | <0.001 | 239 | 34.9 | <0.001 |
| Vomiting (3 or more episodes in day) | 209 | 13.9 | 1682 | 24.3 | <0.001 | 172 | 25.1 | <0.001 |
| **Clinical Laboratory** |  |  |  |  |  |  |  |  |
| Moderate hemoconcentration, no. (%) | 12 | 0.8 | 170 | 2.5 | <0.001 | 21 | 3.1 | <0.001 |
| Severe hemoconcentration, no. (%) | 3 | 0.2 | 48 | 0.7 | 0.040 | 7 | 1.0 | 0.022 |
| Thrombocytopenia, no. (%) | 30 | 2.0 | 422 | 6.1 | <0.001 | 241 | 35.2 | <0.001 |
| Leukopenia, no. (%) | 299 | 19.9 | 1360 | 19.7 | 0.831 | 520 | 75.9 | <0.001 |
| Median platelet count (x 10^3), (range) | 214 | (23.0 - 635.0) | 233 | (10.0 - 727.0) | <0.001 | 122 | (10.0 - 511.0) | <0.001 |
| Median white blood Cell (x 10^3), (range) | 6.7 | (1.8 - 22.0) | 8.3 | (1.0 - 53.0) | <0.001 | 3.3 | (1.0 - 24.0) | <0.001 |

*P-value for the difference in proportion or median between PCR-positive chikungunya and other AFI cases using the Chi-square test or Mann-Whitney Wilcoxon test, respectively.

**P-value for the difference in proportion or median between PCR-positive chikungunya and PCR-positive dengue cases using the Chi-square test or Mann-Whitney Wilcoxon test, respectively.

† Signs of poor circulation included report of pale cold skin, and/or having cyanosis.
