# Supplementary material for: Distinguishing patients with laboratory-confirmed chikungunya from dengue and other acute febrile illnesses, Puerto Rico, 2012–2015
Source: PLoS Negl Trop Dis. 2019 Jul 22;13(7):e0007562. doi: 10.1371/journal.pntd.0007562 (PMC6645456; doi:10.1371/journal.pntd.0007562)
Supplement: S2 Table — (DOCX) [file pntd.0007562.s003.docx]

**S2 Table.** Predictors of laboratory-confirmed chikungunya versus all other acute febrile illnesses (AFI) and laboratory-confirmed dengue by timing of presentation.

| **Timing of presentation in days post-illness onset (DPO)** | **Positive Predictors for Laboratory-Confirmed Chikungunya** | | | | | | | | | **Negative Predictors for Laboratory-Confirmed Chikungunya** | | | | | | | | | | | | | |
| --- | --- | --- | --- | --- | --- | --- | --- | --- | --- | --- | --- | --- | --- | --- | --- | --- | --- | --- | --- | --- | --- | --- | --- |
|  | Joint pain | Red, swollen joints | Muscle, bone, or back pain | Skin rash | Pruritic skin | Facial and/or neck erythema | Red conjunctiva | Any bleeding | Irritability | Thrombocytopenia | Leukopenia | Cough | Rhinorhea | Sore throat | Diarrhea | Abdominal pain | Nausea | Anorexia | Poor circulation | Dizziness | Head ache | Eye pain |  |
| **Any DPO** |  |  |  |  |  |  |  |  |  |  |  |  |  |  |  |  |  |  |  |  |  |  |  |
| All AFIs | X | X^*^ | X | X |  | X^‡^ | X | X^‡^ |  | X | X^†^ | X | X | X | X | X |  | X | X |  | X |  |  |
| Dengue | O | O |  | O |  |  |  | O | O | O | O | O |  |  | O |  | O |  | O | O | O | O |  |
| **<3 DPO** |  |  |  |  |  |  |  |  |  |  |  |  |  |  |  |  |  |  |  |  |  |  |  |
| All AFIs | X | X^**^ | X | X |  | X^**^ |  | X^‡^ |  | X |  | X | X | X | X | X | X | X | X |  |  |  |  |
| Dengue | O | O |  | O^£^ |  |  |  |  |  | O | O |  |  |  |  |  | O |  |  |  |  |  |  |
| **3-5 DPO** |  |  |  |  |  |  |  |  |  |  |  |  |  |  |  |  |  |  |  |  |  |  |  |
| All AFIs |  | X |  | X | X |  | X |  |  | X |  |  | X | X |  |  |  |  | X |  |  |  |  |
| Dengue |  | O |  |  | O |  |  |  |  | O | O | O |  |  |  |  | O |  |  |  |  |  |  |

X = comparison group was all other AFI; O = comparison group was RT-PCR positive dengue cases.

*Positive predictor of chikungunya in all groups except children < 5 years old.

† Negative predictor of chikungunya only among children <20 years old.

‡ Positive predictor of chikungunya only among children <5 years old.

** Positive predictor of chikungunya only among adults 20 years old or older.

£ Positive predictive of chikungunya only among children 5 - 19 years old.
